# Clinical chimeric antigen receptor T‐cell therapy: a new and promising treatment modality for glioblastoma

**DOI:** 10.1002/cti2.1331

**Published:** 2021-08-11

**Authors:** Michael P Brown, Lisa M Ebert, Tessa Gargett

**Correction to:***Clin Trans Immunol* 2019; **8**: e1050. doi: https://doi.org/10.1002/cti2.1050. Published online 20 May 2019.

The authors inadvertently excluded a funding body in the Acknowledgments section. The corrected version appears below:

**ACKNOWLEDGMENTS**

We acknowledge support from the Cancer Council SA Beat Cancer Project Hospital Research Support Package, a Cancer Council Early Career Research Fellowship, the Health Services Charitable Gifts Board (Adelaide) and The Hospital Research Foundation.

The authors apologise for this error.

